# Expression Profiling without Genome Sequence Information in a Non-Model Species, Pandalid Shrimp (*Pandalus latirostris*), by Next-Generation Sequencing

**DOI:** 10.1371/journal.pone.0026043

**Published:** 2011-10-10

**Authors:** Ryouka Kawahara-Miki, Kenta Wada, Noriko Azuma, Susumu Chiba

**Affiliations:** 1 Genome Research Center, NODAI Research Institute, Tokyo University of Agriculture, Setagaya-ku, Tokyo, Japan; 2 Faculty of Bioindustry, Tokyo University of Agriculture, Abashiri, Hokkaido, Japan; Argonne National Laboratory, United States of America

## Abstract

While the study of phenotypic variation is a central theme in evolutionary biology, the genetic approaches available to understanding this variation are usually limited because of a lack of genomic information in non-model organisms. This study explored the utility of next-generation sequencing (NGS) technologies for studying phenotypic variations between 2 populations of a non-model species, the Hokkai shrimp (*Pandalus latirostris*; Decapoda, Pandalidae). Before we performed transcriptome analyses using NGS, we examined the genetic and phenotypic differentiation between the populations. Analyses using microsatellite DNA markers suggested that these populations genetically differed from one another and that gene flow is restricted between them. Moreover, the results of our 4-year field observations indicated that the egg traits varied genetically between the populations. Using mRNA extracted from the ovaries of 5 females in each population of Hokkai shrimp, we then performed a transcriptome analysis of the 2 populations. A total of 13.66 gigabases (Gb) of 75-bp reads was obtained. Further, 58,804 and 33,548 contigs for the first and second population, respectively, and 47,467 contigs for both populations were produced by *de novo* assembly. We detected 552 sequences with the former approach and 702 sequences with the later one; both sets of sequences showed greater than twofold differences in the expression levels between the 2 populations. Twenty-nine sequences were found in both approaches and were considered to be differentially expressed genes. Among them, 9 sequences showed significant similarity to functional genes. The present study showed a *de novo* assembly approach for the transcriptome of a non-model species using only short-read sequence data, and provides a strategy for identifying sequences showing significantly different expression levels between populations.

## Introduction

Phenotypic variation and its adaptive significance have attracted considerable attention from evolutionary biologists. The phenotype is understood to be shaped by interactions between genetic and environmental effects [1]–[3]; however, it can be difficult to clarify whether phenotypic variation is a plastic response to environmental influences or is a consequence of genetic variation. Elucidation of the genetic and environmental effects on a phenotype has been often accomplished by controlled experimental manipulations (e.g., [4]–[7]) or by the identification of cases in which phenotypic variation is caused by genetic adaptation and phenotypic plasticity working in opposite directions [2]. Furthermore, recent statistical analyses have succeeded in revealing genetic changes in certain traits by using decadal data sets (e.g., [8]–[10]). While these approaches are reliable for clarifying the evolution of relevant traits, the organisms or situations amenable to these types of analyses are limited. Meanwhile, approaches using molecular techniques are ideal for detecting genetic variation and genotype by environment interaction (e.g., [11]–[13]). However, genomic information remains scarce except for some model systems, and an enormous workload would be required to establish a molecular biological analysis by applying classical long-read technologies to non-model organisms lacking any genome information.

The development of next-generation sequencing (NGS) technologies allows the acquisition of more sequence data per run at a substantially lower cost than in long-read technologies [14]. Yet, because of the short read lengths, the application of NGS technologies has generally been restricted to model organisms for which the genome sequences are already known. However, recent algorithmic and experimental advances have made it possible to succeed at *de novo* sequence projects [15], [16]. In particular, transcriptome analyses in which complexity is reduced rather than those in genomic one, are receiving attention as they are likely to be suitable for discovering some expressed genes, single nucleotide polymorphisms (SNPs), and microsatellite regions in non-model organisms. For example, Gibbons et al. [17] suggested the utilities of short-read sequencing for evolutionary studies on tropical disease vectors *Aedes aegypti* and *Anopheles gambiae*. Furthermore, species differentiation of crow species *Corvus corone* and *Corvus cornix* was detected through differences in their gene expression profiles, whereas the use of several DNA markers failed to detect this differentiation [18]. In mammals, transcriptome analyses in the Antarctic fur seal *Arctocephalus gazella* using mRNA derived from skin tissues detected several thousand putative microsatellite loci and SNPs [19]. These pioneering studies have suggested that NGS provides a massive amount of useful information for population genetics in non-model organisms. Although NGS technologies have never applied to generating gene expression profiles associated with specific traits, the application of NGS may introduce a new perspective to evolutionary study on phenotypic variation in wild organisms.

**Figure 1:**
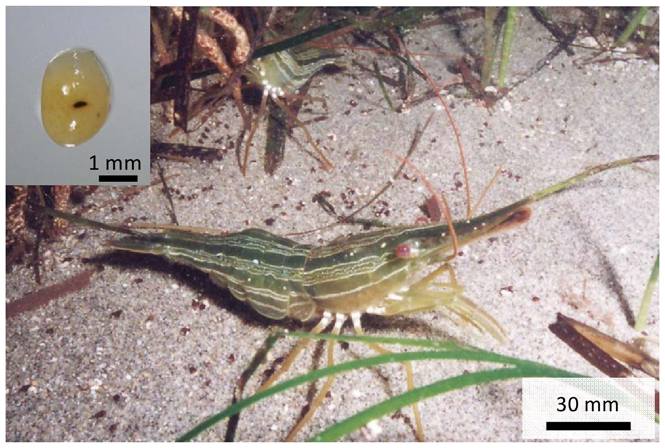
A *Pandalus latirostris* (Hokkai shrimp) adult and an egg. The black spot on the egg (left above corner) is one of the eyes, and the dark-white region is the undeveloped embryo of the shrimp.

**Figure 2:**
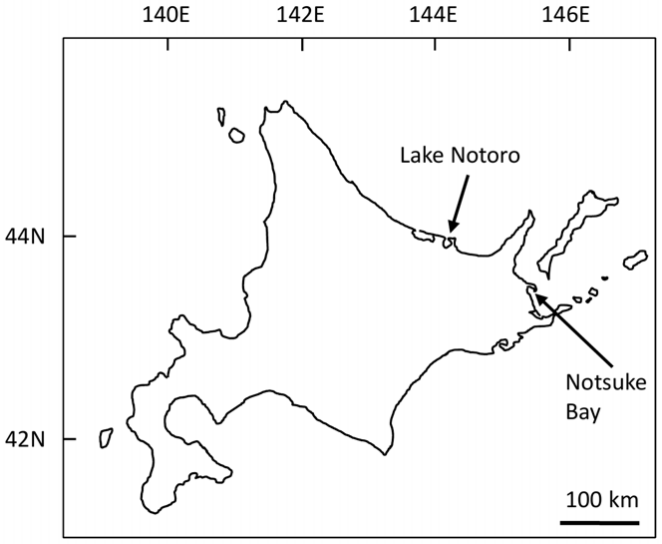
Map showing the locations of the study lagoons, Notsuke Bay and Lake Notoro, in Hokkaido. Seagrasses (*Zostera* spp.) are the habitat of Hokkai shrimps and are distributed only within these lagoons.

Here, we explored the utility of NGS technologies for studying phenotypic variations between 2 populations of the Hokkai shrimp *Pandalus latirostris* (Decapoda, Pandalidae), which lacks genome and transcriptome sequence information (Fig. 1). In general, high gene flow is expected within a latitudinal range of localities in marine organisms because of their high migration and dispersal abilities. Although the geographical distance between the populations in this study is only about 100 km at a direct distance (Fig. 2), it remains possible that the shrimps in each population have locally adapted since they are established in discrete habitats in seagrass areas inside lagoons and do not have a planktonic larval period [20]. It is an important aspect to demonstrate whether there is phenotypic variation between the populations for the conservation and fishery management of this shrimp. While this species is one of the pandalid shrimps endemic to northern coastal Japan and Primorye, Russia [21], they are heavily exploited because of their scarcity value [22]–[24]. For example, the mean annual landings in Hokkaido, Japan from 2004 to 2008, were only 244.2 T but accounted for over 6 million USD [25]. However, their abundance has gradually decreased or become unstable, at least in Japan [25], and transplantation between populations is planned without the consideration of their genetic background in some populations where the fishing yields are declining severely (Hokkaido government, unpublished information). Therefore, the population divergence in their genetic traits must be examined in order to avoid conducting an unfavorable genetic disturbance.

In this study, we first examined the genetic differentiation between the 2 populations using microsatellite genetic markers, which allowed us to estimate the degree of gene flow between the populations. Second, we compared body size at maturity, egg number, and egg size as typical quantitative fitness-related traits [26] by conducting field observations over the course of 4 years. The results of the 2 approaches showed that these populations genetically differed from one another and indicated that the egg traits varied genetically between the populations. Finally, we conducted a transcriptome analysis using mRNA extracted from the ovaries of 5 females in each population and examined whether any genetic differences were observed. The analysis was successful in detecting genes that were differentially expressed between the 2 populations and showed a *de novo* assembly approach for analyzing the transcriptome of a non-model species using only short-read sequence data.

## Results

### Genetic differentiation using microsatellite markers

The 2 populations of *P. latirostris* used in this study were collected at the lagoons Notsuke Bay (NTK) and Lake Notoro (NTR) in Hokkaido, Japan (Fig. 2). The allele numbers and observed and expected heterozygosities (*H_O_* and *H_E_*) of 8 microsatellite loci in NTK and NTR are shown in Table S1. No locus showed significant deviation from Hardy-Weinberg equilibrium and no pairs of loci showed significant linkage disequilibrium, with the significance level set at 0.01 for each population. These results suggested that population genetic analysis using these markers leads to reliable results. The pairwise *F_ST_* value between NTK and NTR was 0.021, with *p* = 0.000 for significant deviation from zero, suggesting significant genetic distance between the 2 populations. An individual-based assignment test indicated that the most plausible number of the source population was 2. The proportion of membership of assumed populations 1 and 2 was 0.681 and 0.319 in NTK and 0.288 and 0.712 in NTR, respectively, suggesting genetic heterogeneity between NTK and NTR.

### Comparison of the phenotypes

There was no clear population divergence in body size throughout the observation period. While the body sizes of the NTK shrimp were smaller than those of the NTR shrimp in 2007 (F_1,61_ = 4.20, *p* = 0.04), the NTK shrimp had a larger body size in 2008 (F_1,34_ = 4.85, *p* = 0.03). No significant differences in size were detected in 2009 (F_1,55_ = 0.00, *p* = 0.97) and in 2010 (F_1,62_ = 0.02, *p* = 0.88).

**Figure 3:**
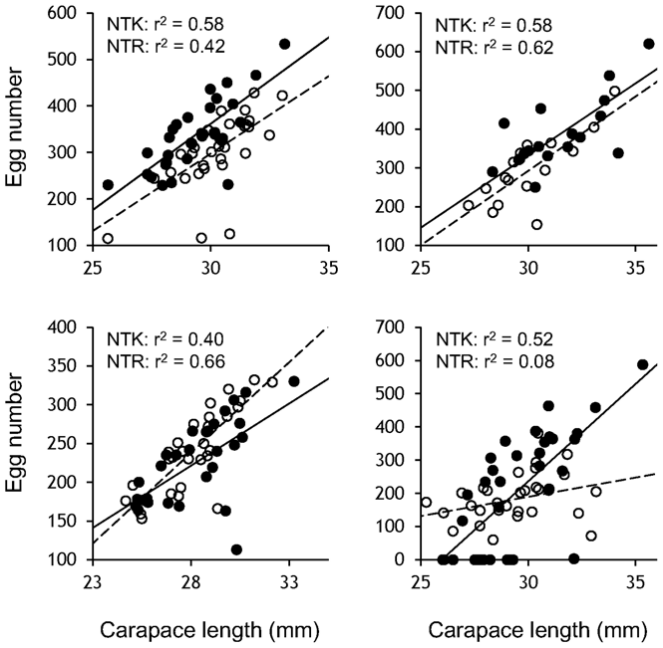
Relationships between female body size and the number of eggs in Hokkai shrimp. The data for the NTK and NTR populations are shown with filled and open circles, respectively. The solid and dashed lines denote the regression lines between the female carapace length and egg number in the NTK and NTR populations, respectively. The egg number significantly increased with female body size except in the case of the NTR population in 2010.

For the NTK and NTR populations, the egg number generally increased with body size. Moreover, there was no clear population divergence in the relationship between the 2 measurements (Fig. 3). The intercept of the egg number of the NTK population was higher than that of NTR one in 2007 (t = 4.47, df = 60, *p* = 0.00), indicating that the NTK females produced more eggs than the NTR ones at the same body size. However, the relationship did not differ between populations in 2008 (t = 1.74, df = 33, *p* = 0.09) and 2009 (t = −1.83, df = 54, *p* = 0.07). Although the breeding season had finished before the observations in 2010, some of the females in both populations did not carry eggs, and the egg number did not significantly increase for the NTR population in 2010 (*p* = 0.12). Because the slopes of the regression lines in 2010 were not statistically equal, we did not compare the egg number between populations.

**Figure 4:**
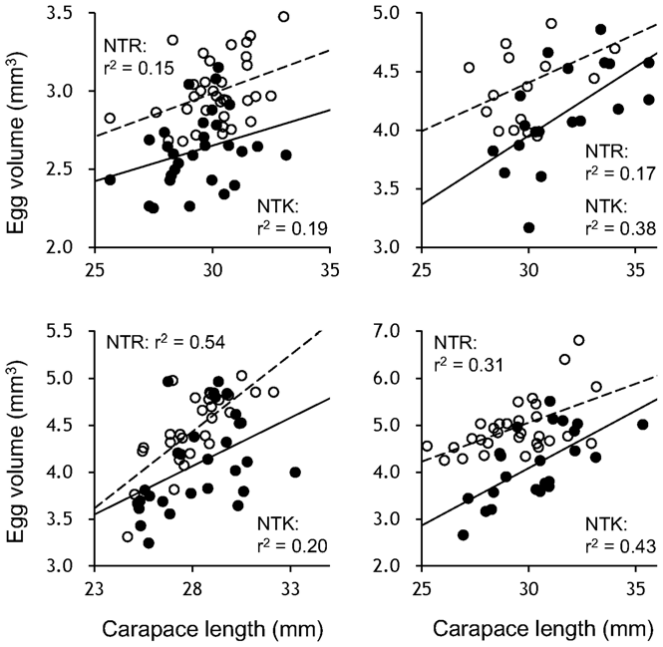
Relationships between female body size and egg size in Hokkai shrimp. The data for the NTK and NTR populations are shown with filled and open circles, respectively. The solid and dashed lines denote regression lines between female carapace length and egg size in the NTK and NTR populations, respectively. The egg sizes significantly increased with female body size in both populations throughout the observations.

We detected a clear population divergence in the relationship between body size and egg size (Fig. 4). We found that visible eyespot on the eggs of both the NTK and NTR shrimp, but the embryos were not yet clearly defined (Fig. 1). Throughout the observation period, however, the egg sizes of the NTK females were significantly smaller than those of NTR ones (t = −5.62, df = 60, *p* = 0.000 in 2007; t = −3.47, df = 33, *p* = 0.000 in 2008; t = −3.85, df = 54, *p* = 0.000 in 2009; t = −6.22, df = 49, *p* = 0.000 in 2010), indicating that the NTK females produced smaller eggs than the NTR ones at the same body size.

### Transcriptome sequencing and assembly

We sequenced 4 lanes (2 lanes in 2 sequence runs) of the cDNA library from the NTK and NTR populations that were maintained in the laboratory (see Materials and Methods), and 13.66 Gb of 75-bp reads (7.19 Gb and 6.64 Gb for the NTK and NTR populations, respectively) were obtained. Sequence data were deposited in the DDBJ Read Archive (DRA) (Accession #: DRA000399).

**Figure 5:**
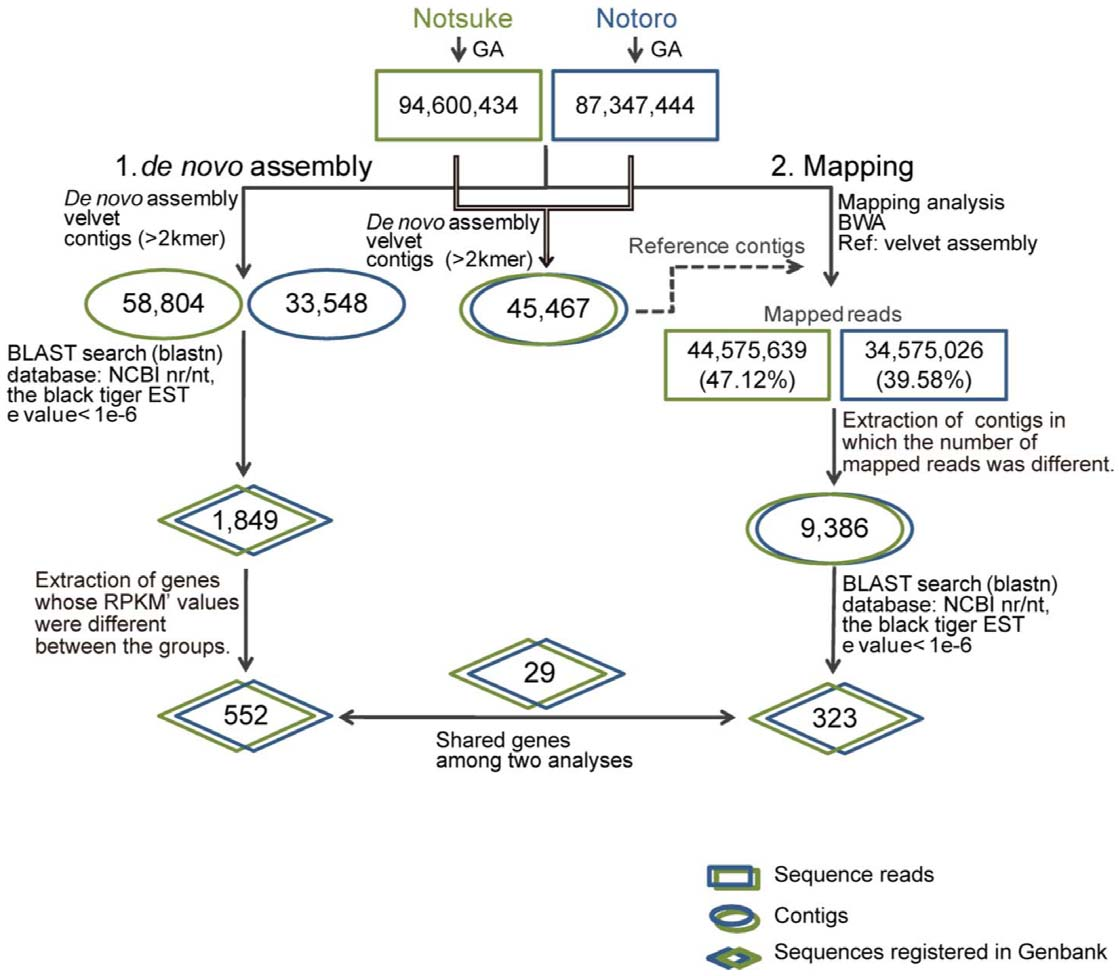
Bioinformatics workflow and results of the single steps of the workflow (assembly and expression analysis).

Reads were then used for transcriptome assembly and gene expression analysis as described in the “Materials and Methods” section. The bioinformatics workflow is summarized in Figure 5, along with the results of the single steps of the workflow (assembly and expression analysis).

The results of the *de novo* assembly are summarized in Table 1. We assembled varying amounts of sequence reads for the 2 populations of Hokkai shrimp. We obtained 58,804 contigs for NTK, 33,548 contigs for NTR, and 47,467 contigs for both populations. The total amount of sequence reads that contributed to the assemblies was 3.5 Gb for NTK (49.75%), 2.7 Gb for NTR (40.95%), and 6.0 Gb (43.74%) for both populations, respectively (Fig. S1 A). The number of assembled contigs and the number of reads used in the assemblies were dependent on the starting number of reads used as input to the Velvet program (Fig. S1 B, C). Although the number of assembled contigs and N50 seemed to plateau as the number of reads increased, the total amount of the reads used in the assemblies did not seem to plateau as reads from additional lanes were used (Fig. S1 B–D). The numbers and total sequence amounts of the assembled contigs were also dependent on *k*-mer length (Fig. S1 E–G). There were positive relationships between *k*-mer length and median contig coverage depth and between *k*-mer length and median contig length, whereas there was a negative relationship between *k*-mer length and contig number, which is consistent with a previous study [17].

**Table 1:** Summary of *de novo* assembly using Velvet.

Group	Lane #	Read #	*k*-mer	Contig #	N50	Max. Len.	Med. Len.	Med. Cov
NTR	1	46,611,502	63	24,805	356	3658	430	89.4
	1	40,735,942	69	6,111	397	3582	290	127.3
	2	87,347,444	63	33,548	414	5837	272	79.4
NTK	1	51,047,240	61	36,554	419	5755	271	61.4
	1	43,553,194	63	28,966	394	6995	269	61.3
	2	94,600,434	61	58,804	420	5565	260	58.6
All	4	181,947,878	61	45,467	493	6434	309	109.4

Abbreviations: “Lane #”: number of sequencing lanes used as input in the assembly; “Read #”: number of sequence reads used as input in the assembly; “*k*-mer”: required length of identical match between two sequence reads by the Velvet software; “Contig #”: number of contigs produced by the assembly; “Max. Len.”: maximum length of contigs; “Med. Len.”: median length of contigs; and “Med. Cov.”: median coverage depth of contigs.

### Expression analysis

**Figure 6:**
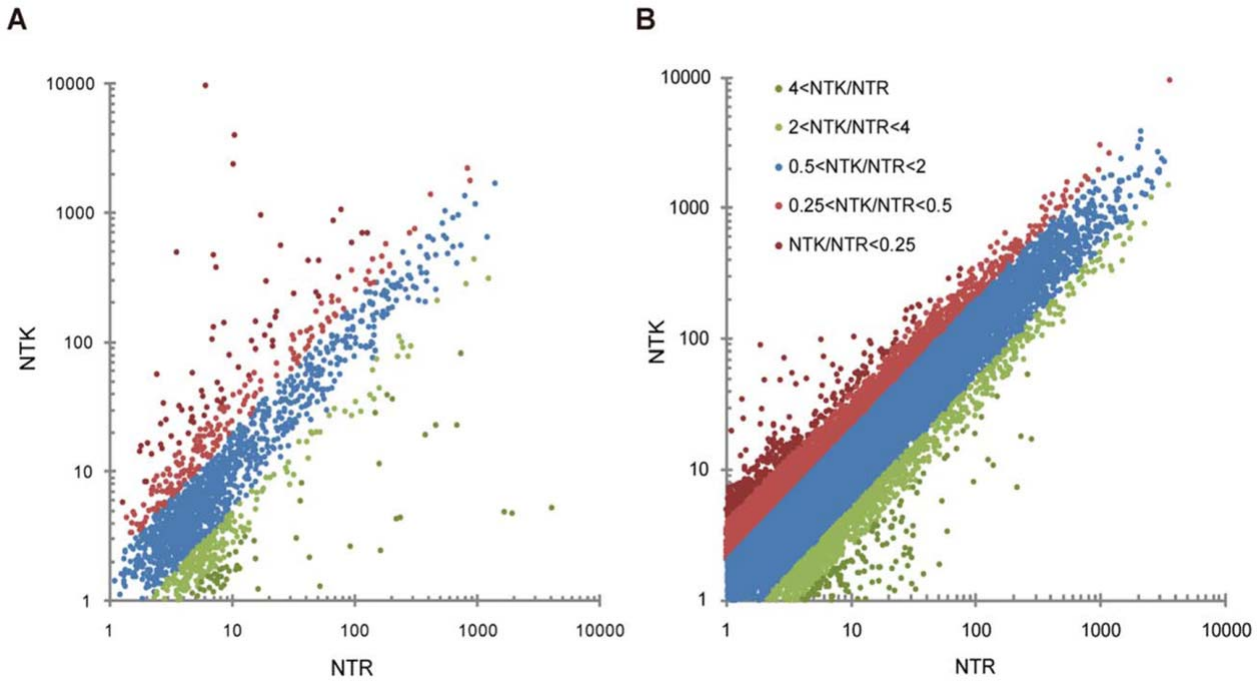
Scatter plot for normalized expression levels for all contigs in NTK and NTR study populations. (A) For the *de novo* assembly-based strategy, 1,849 contigs were assigned to a unique gene in the NCBI nr/nt database and were used for the scatter plot, with 552 contig pairs showing more than twofold differences in expression. (B) For the mapping-based strategy, all reads from each of the 2 experimental groups were aligned to the Hokkai shrimp representative assembly, and the normalized number of the mapped reads in each group was used for the scatter plot. As a result, 11,583 contig pairs showed a greater than twofold difference in expression levels.

We performed expression analysis using 2 different assembly strategies as described in the “Materials and Methods” section and as summarized in Figure 5. In the *de novo* assembly-based strategy, we assembled the sequence reads from each population and used BLASTN to assign the contigs to the NCBI nr/nt database and the black tiger prawn EST database. Among the contigs that were assigned to unique genes in the NCBI nr/nt database, 1,849 contigs pairs were found in both populations and retained in the dataset. For each contig pair, the normalized expression level was compared each other, and 552 contig pairs showed greater than twofold differences in the expression levels (Fig. 6, Table S2).

In the mapping-based strategy, the Hokkai shrimp assembly was computed from all sequence reads, and the resultant assembly contained 45,467 contigs that were used as a reference in the mapping analysis. All the reads from each population were aligned to the Hokkai shrimp assembly using BWA. The 44,575,639 reads (47.1%) for the NTK population and the 34,575,026 reads (39.6%) for the NTR one were subsequently mapped to the assembly (Fig. 5). The percentage of the number of reads mapped to the assembly as references was largely concordant with the percentage of the number of reads used in the assemblies (49.8% for the NTK population and 40.95% for NTR one). For each contig of the assembly, the number of mapped reads was compared between the NTK population and the NTR one (Fig. 6). As a result, the expression levels of 11,583 contig pairs were found to be more than twofold different between the 2 populations. These differentially expressed contigs were submitted to a BLASTN search using the NCBI nr/nt database and the black tiger prawn EST database (e-value <1e-6), and 702 contig pairs were assigned to known sequences in the databases (Table S3). Differentially expressed gene lists from the 2 approaches were compared, and 52 sequences were found to be present in both lists. Among them, 29 showed concordance in either increasing or decreasing for both approaches, and were therefore considered to be differentially expressed genes (Table 2). Fisher's exact test showed that 16 of the genes were statistically significant.

**Table 2:** Expression changed genes list.

Accession #	Description	NTK/NTR (mapping)	NTK/NTR (assembly)
AF169742.1	*Palaemon serenus* large subunit ribosomal RNA gene, partial sequence	2.10	3.31 **
AY466445.1	*Macrobrachium rosenbergii* heat shock protein 70 mRNA, complete cds	2.73 **	2.10 **
BT083252.1	*Anoplopoma fimbria* clone afim-evh-518-317 Trafficking protein	2.10	2.330
DQ660140.1	*Macrobrachium nipponense* heat shock cognate 70 (hsc70) mRNA, complete	2.02 **	4.95 **
DW677957.1	HC-H-S01-0530-LF heat-induced haemocyte cDNA library *Penaeus monodon*	2.60 **	6.18 **
EB390061.1	OV-N-S01-0692-W Ovarian *Penaeus monodon* cDNA library *Penaeus monodon*	2.11	2.130
EZ420767.1	TSA: *Haliotis asinina* HasCL137Contig1, mRNA sequence	2.34 **	13.71 **
GO068233.1	PmTwI22F06 PmTwI *Penaeus monodon* cDNA 3′, mRNA sequence	2.10	2.990
GQ487506.1	*Pandalus montagui* voucher KC3144 18S ribosomal RNA gene, partial	2.14 **	381.58 **
HO000133.1	HC-H-S01-0103-LF heat-induced haemocyte cDNA library *Penaeus monodon*	2.95 **	4.64 **
X95444.1	*S.lividans* Rho gene	69.25 **	4.82 **
XM_001459489.1	*Paramecium tetraurelia* hypothetical protein (GSPATT00024860001)	2.51	3.130
XM_002057372.1	*Drosophila virilis* GJ17070 (Dvir\GJ17070), mRNA	4.26 **	3.500
XM_002807980.1	PREDICTED: *Macaca mulatta* tubulin alpha-1C chain-like (LOC710110),	3.43 *	3.100
XM_536543.2	PREDICTED: *Canis familiaris* similar to Heat shock cognate 71 kDa	2.41 **	2.73 **
Y08260.1	*M.musculus* mRNA for CPEB protein	2.00	2.080
AC192469.4	*Pan troglodytes* BAC clone CH251-585B11 from chromosome 20, complete	0.49 **	0.30 **
AF100986.1	*Penaeus monodon* actin 1 (act1) mRNA, complete cds	0.11 **	0.09 **
AI253853.1	AIMS-P.mon56 Giant tiger prawn pleopod cDNA library *Penaeus monodon*	0.44 **	0.44 **
DW042952.1	HC-V-S01-0457-LF Hemocyte - *Vibrio harveyi* infected library *Penaeus monodon*	0.34 **	0.46 **
DW404916.1	ES-N-S02-0058-W *Penaeus monodon* Eyestalk cDNA library *Penaeus monodon*	0.29 **	0.45 **
EB389985.1	OV-N-S01-0563-W Ovarian *Penaeus monodon* cDNA library *Penaeus monodon*	0.46	0.45 **
XM_001980837.1	*Drosophila erecta* GG17398 (Dere\GG17398), mRNA	0.46 **	0.450
XM_001999423.1	*Drosophila mojavensis* GI23057 (Dmoj\GI23057), mRNA	0.46 **	0.49 **
XM_002606105.1	*Branchiostoma floridae* hypothetical protein, mRNA	0.42 **	0.460
EF364538.1	*Macrobrachium rosenbergii* clone G male reproductive-related protein	0.08 **	0.20 *
GE614960.1	OV-N-S01-0922 *Penaeus monodon* ovary *Penaeus monodon* cDNA 5′, mRNA	0.49 **	0.38 **
GO066557.1	PmTwI04E11.scf PmTwI *Penaeus monodon* cDNA 3′, mRNA sequence	0.40 **	0.49 *
GO070224.1	PmTwI44F11.scf PmTwI *Penaeus monodon* cDNA 3′ similar to B Chain B,	0.37 **	0.44 **

*p<0.05,

**p<0.01.

Among the differentially expressed genes, the expression levels increased in 16 sequences and decreased in 13 sequences in the NTK population relative to the NTR one. Out of the 16 sequences with an increased expression level, 5 sequences showed significant similarity to EST sequences, while 2 sequences were genes that encoded unannotated hypothetical proteins. Among the remaining sequences, 2 sequences were ribosomal RNA genes, 3 were members of heat shock protein 70-kDa family (*HSP70*), 3 were related to biosynthetic processes, and 1 was related to a cellular component. Out of the 13 sequences with decreased expression levels, 7 sequences showed significant similarity to EST sequences, 1 was a genomic sequence, and 3 were genes encoding unannotated hypothetical proteins. Among the remaining 2 sequences, 1 sequence was related to a cellular component, and 1 was related to reproduction.

## Discussion

### Genetic and phenotypic divergence between populations

The analyses using microsatellite DNA markers suggested that the NTK and NTR populations genetically differed from each other, indicating that gene flow is restricted between them. Although gene flow is generally considered to be high in marine invertebrates, a restricted one is reasonable in the case of Hokkai shrimp, since this species becomes established in discrete habitats within an isolated seagrass area and has no planktonic larval period [20]. Even though the genetic differentiation between the 2 populations may be attributable to genetic drift, the differentiation at least occurred in the presence of a reproductive barrier. Thus, the results suggested the potential of local adaptation along with independent evolution in each population.

Population divergence in the body sizes of Hokkai shrimps was not clear in our 4-year observation. Body size at maturation is one of the most important fitness-related traits in animals and is a selected trait under each environment [26]. However, the growth rate of ectothermic marine organisms generally fluctuates with both biological factors such as food availability and physical factors such as ambient temperature. Previous studies have demonstrated that somatic growth of Hokkai shrimp also fluctuates between years because of water temperature in their habitats [20], [27]. Moreover, since Hokkai shrimps have been annually harvested from each population, fishing may have influenced the body size as well. Therefore, our results suggest that the difference in their growth rate is larger within than between populations under the natural environment.

We observed an interesting population divergence in the egg traits. The optimal number of eggs produced by a single female can be genetically determined in each environment. To date, a number of studies have demonstrated that a trade-off exists between egg size and number, and that this antagonistic relationship must be optimized by natural selection (e.g., [26]–[29]). The observed unclear relationship between body size and egg number in the Hokkai shrimp suggests that maternal conditions such as temporal physiological status strongly affected the egg number and that the egg number relative to body size could fluctuate in each population. In contrast, we detected that the egg sizes of the NTK females were consistently smaller than those of NTR ones throughout the observations. Although the comparative study on the physical and biological environments of these lagoons is limited, it cannot be assumed that a particular environmental factor in the lagoons is the only factor regulating egg sizes. Therefore, these results suggest that the egg sizes are regulated by genetic factors rather than environmental ones. Although the genotype by environment interaction may differ between these egg traits [30], our field observations imply that genetic differences in the egg traits exist between the 2 populations of Hokkai shrimp.

### Differentially expressed genes in the phenotypically differentiated shrimp populations

In this study, a transcriptome analysis was performed to obtain genomic information regarding the egg-related traits, which is diversified in the 2 populations of Hokkai shrimp. Although there have been several studies on the key determinants of egg phenotypes, the underlying molecular mechanisms are not yet fully understood. In mammals, it has been reported that oocyte size is not independently controlled but is determined by strong adhesion between the oocyte plasma membrane and the inner surface of the zona pellucida [31]. Han et al. (2009) found positive correlations between the inter-individual variation in follicle vitellogenin/very low density lipoprotein receptor (VTG/VLDL) mRNA expression and variation in egg mass in the zebra finch (*Taeniopygia guttata*) [32]. In the silkworm *Bombyx mori*, it is hypothesized that egg size is determined by the egg size-determining (*Esd*) gene located on the W chromosome [33]; however, the presence of *Esd* was not confirmed in a recent study [34]. Recently, Ibarra et al. (2009) demonstrated that VTG levels in the hemolymph of an adult *Penaeus vannamei* shrimp population were genetically and positively correlated to oocyte diameters [35].

The results of our transcriptome analyses allow us to anticipate that some of the identified genes will function in egg development in the Hokkai shrimp. Although the difference in the expression was not statistically significant (Table 2), the *CPEB* gene encodes a cytoplasmic polyadenylation element binding protein — a sequence-specific RNA binding protein that regulates translation during vertebrate oocyte maturation [36]–[39]. Female *CPEB* Knockout mice show vestigial ovaries that are devoid of oocytes, while males display germ cells that are arrested at pachytene [40]. *HSP70* and *HSC70*, which belong to the *HSP70* family, were up-regulated in the NTK population. *HSP70* has been associated with cellular proliferation, differentiation, and cell death [41], [42]. *HSP70* shows expression in interstitial cells and oocytes of maturating ovaries in sea urchins [43].

The list of differentially expressed genes derived from our transcriptome analysis did not include genes encoding proteins that have been reported to be involved in influencing egg traits [35]. The strategy described in this study used 2 approaches, and genes identified in both approaches were selected as differentially expressed. Because this strategy represents a conservative one, false-positive genes were less likely to be retained in the final gene list; however, some potentially egg trait-related genes may have been discarded during the analysis. When the lists of differentially expressed genes from each approach were compared, the agreement between them was very small (Tables S2 and S3). One of the approaches may be more plausible than the other, but it is difficult to say which approach is better because the principle of the programs used in each approach was different: the assembly-based approach used the assembly program BWA, and the mapping-based approach used both BWA and Velvet, a *de novo* assembly program. Validation experiments such as qPCR or microarray would provide some information for resolving this issue. Additionally, in the list of differentially expressed genes, the extent of the differential expression varied between the approaches for some transcripts, including the *Pandalus montagui* voucher KC3144 18S ribosomal RNA gene and the *Streptomyces lividans* Rho gene (Table 2). The differences may be a result of the programs used in each approach, as well as the features (e.g., the way to handle “N” sites for the sequences or the seed length used in the analyses) of the programs. These facts may influence such differences in the expression levels of some transcripts. Further analysis of the gene products, such as proteins and metabolites, which directly influence the phenotype, may provide finer resolution of the molecular mechanisms underlying egg-related traits.

### Conclusion

The present study successfully demonstrated that NGS could be a useful tool for gene expression profiling in a non-model species lacking genomic information. In this study, although the basis for phenotypic differences in egg traits at the gene level is still unknown, the transcriptomic analysis presented here suggests various candidate genes could be differentially expressed between these populations. NGS provides a low cost, labor-saving, and rapid means of transcriptome sequencing and characterization. Recently, *de novo* transcriptome analyses, that is, *de novo* assembly of short reads from mRNA without genome reference, have emerged. Several studies have reported the transcriptome sequencing of various non-model species using NGS technologies. Although most of these studies are based on the long-read sequence data using 454 pyrosequencing or employ a hybrid approach, the *de novo* assembly of transcriptomes using short reads (Illumina or SOLiD) has also received attention because of its relatively low cost [17], [44]–[46]. In this study, we presented a *de novo* assembly approach for the transcriptome of a non-model species using only short-read sequence data and showed a strategy for identifying sequences with different expression levels between the 2 populations of Hokkai shrimps. The strategy of expression analysis described here can be potentially used for any species. Although currently we can only speculate on the functions of the candidate genes producing the population divergence in the egg traits of the Hokkai shrimp, further detailed analyses of our transcriptome data could contribute towards the development of molecular studies on Hokkai shrimp and may accelerate functional genomic studies of reproduction-related traits. Therefore, we conclude that NGS technologies will provide new insights into the evolutionary study of phenotypic variation in wild organisms.

## Materials and Methods

### Samples

*P. latirostri* was collected at the lagoons Notsuke Bay (NTK) and Lake Notoro (NTR) in Hokkaido, Japan, between mid-October and early November for 4 years from 2007 to 2010 (Fig. 2). These lagoons are geographically close to each other (Fig. 2). The water temperature of these lagoons shows a similar seasonal change [27], [47], and the salinity of both lagoons is about 30 psu [48], [49], although these environmental factors fluctuate annually. We trawled a bottom-trawl net in seagrass areas at each lagoon and collected over 1000 shrimp in each sampling. The sampling procedures for NTK and NTR followed descriptions by Mizushima (1986) and Nishihama et al. (1997), respectively [50], [24]. We estimated the age of the shrimp from the size frequency distributions of collected individuals at each sampling using the common cohort separation procedure [21], [27]. Hokkai shrimp is a protandrous (male-first) sex-changing shrimp, and the sex change from male to female generally occurs at age 2 in both populations [22], [27].

All necessary permits were obtained for the described field studies. Collections of Hokkai shrimps in harvested areas of NTK and NTR were permitted by Hokkaido government, Japan.

### Genetic differentiation using microsatellite markers

We tested the genetic differentiation between the NTK and NTR populations using neutral genetic markers. We randomly chose 40 individuals from NTK ones and 46 from the NTR ones collected at the sampling in 2010 and used 8 microsatellite markers (*Pal-1*, *Pal-2*, *Pal-3*, *Pal-144*, *Pal-203*, *Pal-152*, *Pal-178*, and *Pal-216*) that had been previously developed for this species to examine their genetic differentiation [51]. Genomic DNA was extracted from the legs of ethanol-fixed shrimp using the DNeasy Blood & Tissue Kit (Qiagen) DNA isolation protocol for animal tissue. The PCR amplification was carried out using Qiagen Multiplex PCR Kit (Qiagen) in a total volume of 10 µL, in accordance with the manufacturer's instructions. The thermal profile included a pre-cycling denaturation at 95°C for 15 min, followed by 35 cycles each of denaturation at 95°C for 30 s, annealing at a suitable Ta (see Table S1) for 90 s, and extension at 65°C for 30 s, and then a post-cycling extension at 65°C for 30 min. The PCR products were sized by a CEQ8000 DNA sequencer (Beckman Coulter). For confirming the basic utility of the markers, the *H_O_* and *H_E_* and the deviation of the genotypic frequencies from Hardy-Weinberg expectations were tested with Arlequin ver. 2.000 [52]. Linkage disequilibria were tested with GENEPOP ver. 3.4 [53], [54]. The pairwise *F_ST_* [55] was estimated with Arlequin ver. 2.000 [54] to test the significance of genetic differentiation between the 2 populations. Individual-based assignments were performed in STRUCTURE ver. 2.1 [56], [57], with runs including a burn-in period of 100,000 simulations, followed by 500,000 MCMC simulations and 10 iterations for each *K*, 1–4. The most plausible number of source populations (*K*) was selected by the largest likelihood value (*ln*P*(X|K)*) and the smallest variance of *ln*P*(X|K)*. The average *Q* value for each population represented the proportion of membership of the assumed populations.

### Comparison of the phenotypes

We randomly chose 20–30 females estimated to be at age 2 years from collected samples in each observation and temporarily reared them in a tank (500 L) that was supplied with ambient water pumped from NTR at a constant rate (1 L/min) in the Okhotsk Marine Biology Center (OMBC) of Tokyo University of Agriculture. The NTK and NTR shrimps were fixed with 10% neutral-formaldehyde solution on the same date in mid-November in each year. The females in both populations breed once a year between late August and late September and carry eggs for 9 months until late May of the next year [27], [58]. Therefore, we could treat the eggs as being produced virtually simultaneously between the populations, although the exact oviposition date would have varied within the populations.

We compared the length of the carapaces (the length from the base of the eye to the end of the carapace) of reared females between populations in each year. We used one-way analysis of variance (ANOVA) for these comparisons after confirming homogeneity between the comparison data sets, since all of the data was independent and showed a normal distribution. Moreover, we compared the number and size of eggs between populations in each year. The egg shapes were characterized as oval spheres (Fig. 1), and the egg volume was quantified as egg size. Since these egg traits were a covariate of body size [20], we fitted the regression lines to the relationship between carapace length and egg traits in each population. When the slopes of the regression lines were statistically equal in each comparison, we compared the intercepts of the regression lines using analysis of covariate (ANCOVA). All statistical analyses were performed using R 2.11.1 [59].

### Transcriptome sequencing

We collected 50 size-matched, premature females at age 2 from each population in early June of 2009. To diminish the population differentiation in terms of the females' physiological conditions, we reared them for over 2 months under common water and food environments at OMBC until August 27, the beginning of their breeding season. The females of each population were exposed to a natural photoperiod and fed a commercial diet *ad libitum* in a tank (500 L).

On August 27, we confirmed that all females had matured based on an evaluation of the first and second pleopods, which reflect gonad development [60]. We randomly selected a subset of 5 individuals from each population for transcriptome sequencing. Total RNA was extracted from the ovaries using Trizol (Life Technologies Corp., Carlsbad, CA, USA) according to the manufacturer's protocols and cleaned up using the RNeasy mini kit (Qiagen, Valencia, CA, USA). RNA quality and quantity were assessed on a 2100 Bioanalyzer using the RNA 6000 Nano kit (Agilent technologies, Palo Alto, CA, USA). After the RNA concentration was measured, equal volumes of total RNA from the 5 individuals in each group were pooled and used for library preparation.

Libraries were prepared using an mRNA-Seq Sample Preparation Kit (Illumina Inc., San Diego, CA, USA) according to the manufacturer's instructions. For quality control, an aliquot of the library was cloned into a sequencing vector (TOPO TA Cloning® Kit for Sequencing; Life Technologies Corp.), and 48 clones were sequenced by Sanger sequencing. We found that all sequences were unique and that no duplicates were detected in the analyzed sample. A BLASTN search in the NCBI nr/nt sequence database using these sequences revealed that 29 of the clones had similarities with different sequences and that 13 of these 29 sequences showed similarities with those from Decapoda. According to the sequence results, we confirmed that the derived libraries were suitable for the analysis. The DNA concentration of the cDNA library was measured using a 2100 Bioanalyzer using the DNA 1000 kit (Agilent technologies) and diluted to 10 nM, and a 2 µl aliquot was used to generate clusters on the Illumina Cluster Station using the Paired-End Cluster Generation Kit v2 (Illumina) and sequenced on the GAII using the SBS 36-cycle Sequencing Kit v3 following the manufacturer's instructions. Two lanes per group were sequenced as 75-bp reads, and image analysis and base calling were performed with CASAVA ver.1.6.0 (Illumina) according to the manufacturer's instructions. High-quality sequences (those passing default quality filtering parameters in the Illumina GA pipeline GERALD stage) were retained for further analysis.

### *De novo* assembly

As a preliminary analysis, we performed *de novo* assemblies using 3 programs: Velvet [61], Oases (Schulz and Zerbino, unpublished; available at http://www.ebi.ac.uk/~zerbino/oases/), and ABySS [62], with the sequence reads pooled in each population. The assembly results of the preliminary analysis showed that ABySS produced more and shorter contigs than the other programs. The number of contigs and N50 was better for Velvet, while the greatest length for a contig was observed for an Oases contig. Therefore, on the basis of the results of the preliminary analyis, we decided to use Velvet for further analysis. We assembled all sequence reads from 2 populations of Hokkai shrimp using a set of algorithms, Velvet, which generates assemblies by searching for identical matches of a certain length (referred to as *k*-mer length) between reads [61]. To identify the optimal *k*-mer value, we first assembled our reads using the VelvetOptimizer to optimize the *k*-mer value and the coverage cutoff value based on the number of base pairs in contigs greater than 1 kilobase (kb) in length [http://www.bioinformatics.net.au/software.velvetoptimiser.shtml]. Trimmed reads (66, 68, 70, 72, and 74 bp) were assembled in addition to the assembly using full-length (75 bp) reads to compare their optimized read length based on the number of contigs greater than 1 kb, and the assembly with 75-bp reads was retained for further analysis. Among the assembly, contigs with length greater than 2*k*-mer length were used for further analysis.

### Expression analysis

To identify genes showing differential expression patterns between the 2 populations of Hokkai shrimp, we used 2 approaches: 1 was based on *de novo* assembly, while the other was based on the mapping analysis. In the approach based on *de novo* assembly, we assembled the reads from each of the populations and performed a BLASTN search using the nr/nt database [63] to identify similar sequences for contigs assembled in each population. The hits returned by BLASTN were filtered for matches with significant e-values of smaller than 1e-6. Contigs that could not be assigned to any sequences were submitted to another BLASTN search with the black tiger prawn (*Penaeus monodon*) EST database downloaded from NCBI [http://www.ncbi.nlm.nih.gov/nucest?term=txid6687Organism%3Anoexp]. Contigs assigned to any known sequences were labeled with the accession numbers of the sequences with the highest e-value scores and retained for further analysis. For comparison between the 2 populations of Hokkai shrimp, contigs with accession numbers assigned to both populations were selected. In each selected contig pair with the same accession number, the expression level was determined by the number of reads that made up the contig. According to Mortazavi et al. (2008), the number was normalized based on the total reads used in this experiment and the length of the contigs [64]. Contig pairs whose read numbers were more than twofold different between the populations were selected as candidates for differentially expressed genes.

In the approach based on mapping analysis, we first generated a reference assembly using all sequence reads from both populations of Hokkai shrimp. Next, all reads for each population were mapped using BWA software with default settings [65]. The expression level was determined by the number of mapping sequence reads in each contig. To normalize the expression value, the number of total reads used in the experiment and the length of each assembled contig were used [64]. Length standardization introduces a bias in correlation analysis, thereby leading to artificially high correlation levels and to the rearrangement of data points, with each being shifted by the same amount, as shown in a previous study [18]. However, by increasing the number of decimals in the calculation, this problem was largely solved. Contigs with mapped read numbers showing more than twofold differences between the 2 populations were selected and submitted to a BLASTN search to identify their similar sequences. The hits returned by BLASTN were filtered for matches with significant e-values of smaller than 1e-6. Contigs that could not be assigned to any sequences were submitted to another BLASTN search using the black tiger prawn EST database. Contigs assigned to any known sequences were labeled with the most similar sequence's accession numbers and retained as candidates for differentially expressed genes.

Finally, differentially expressed sequence lists from the 2 approaches were compared and those found in both lists were considered to be differentially expressed. In addition, statistical significance was assigned to each sequence by setting up a Fisher's exact test, in which the number of normalized, mapped reads for each gene was compared to the total number of mapped reads for each population according to a previous study [66].

## Supporting Information

Figure S1 **Assemblies using varying amounts of sequenced read data.** (A) Summary statistics of the assembly and derived contigs. Relationships between the number of starting reads and N50 (B), the number of starting reads and reads used in the assemblies (C), and the number of starting reads and the number of contigs in the assemblies (D) are shown in the graphs. In addition, the relationships between the *k*-mer length and the median coverage of the contigs in the assemblies (E), the *k*-mer length and the number of contigs in the assemblies (F), and the *k*-mer length and the median contig length in the assemblies (G) is shown. Red squares show the data from the first run; blue crystals, the data from the second run; and green triangles, the combined data.
(TIF)

Table S1 **Locus name, primer sequence, annealing temperature (** ***Ta*** **) and number of alleles (** ***NA*** **), observed and expected heterozygosity (** ***H_O_*** **and** ***H_E_*** **) in NTK and NTR for each microsatellite marker.**
(XLS)

Table S2 **Differentially expressed genes extracted using assembly analysis.**
(XLS)

Table S3 **Differentially expressed genes extracted using mapping analysis.**
(XLS)
