# Supplementary figures and images for: Expression Profiling without Genome Sequence Information in a Non-Model Species, Pandalid Shrimp (Pandalus latirostris), by Next-Generation Sequencing
Source: PLoS One. 2011 Oct 10;6(10):e26043. doi: 10.1371/journal.pone.0026043 (PMC3189924; doi:10.1371/journal.pone.0026043)

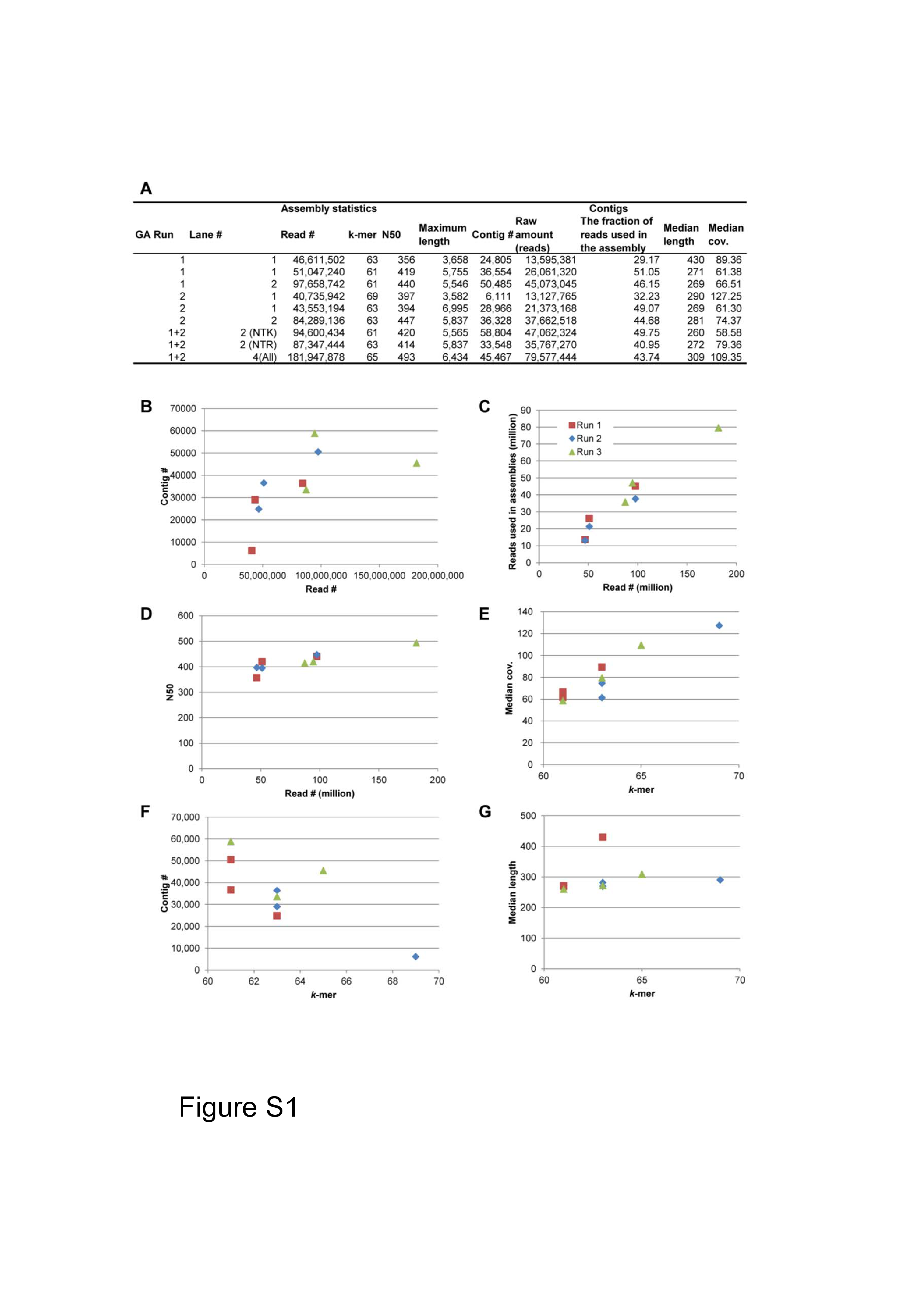

Supplement: Figure S1 — Assemblies using varying amounts of sequenced read data. (A) Summary statistics of the assembly and derived contigs. Relationships between the number of starting reads and N50 (B), the number of starting reads and reads used in the assemblies (C), and the number of starting reads and the number of contigs in the assemblies (D) are shown in the graphs. In addition, the relationships between the k-mer length and the median coverage of the contigs in the assemblies (E), the k-mer length and the number of contigs in the assemblies (F), and the k-mer length and the median contig length in the assemblies (G) is shown. Red squares show the data from the first run; blue crystals, the data from the second run; and green triangles, the combined data. (TIF) [file pone.0026043.s001.tif]
